# Night-Air

**Published:** 1858-09

**Authors:** 


### NIGHT-AIR.

DURING the months of September and October, throughout the United States, wherever there are chills, and fever and ague, intermittents, or the more deadly forms of fever, it is a pernicious, and even dangerous practice, to sleep with the outer doors or windows open; because miasm, marsh emanations, the product of decaying vegetation—all of which are different terms, expressing the same thing—is made so light by heat, that it ascends at once towards the upper portion of atmospheric space, and is not breathed during the heat of the day, but the cool nights of the Fall of the year condense it, make it heavy, and it settles on the ground, is breathed into the lungs, incorporated into the blood; and if in its concentrated form, as in certain localities near Rome, it causes sickness and death within a few hours. The plagues which devastated Eastern countries in earlier ages, were caused by the concentrated emanations from marshy localities, or districts of decaying vegetation; and the common observation of the higher class of people was, that those who occupied the upper stories, not even coming down stairs for market supplies, but drew them up by ropes attached to baskets, had entire immunity from disease, for two reasons, the higher the abode, the less compact is the deadly atmosphere, besides, the higher rooms in a house, in summer, are the warmer ones, and the miasm less concentrated. The lower rooms are colder, making the air more dense. So, by keeping all outer doors and windows closed, especially the lower ones, the building is less cool and comfortable, but it excludes the infectious air, while its warmth sends what enters through the crevices immediately to the ceilings of the rooms, where it congregates, and is not breathed; hence is it that men who entered the bar-room and dining-saloons of the National Hotel, remaining but a few brief hours, were attacked with the National Hotel Disease, while ladies who occupied upper rooms, where constant fires were burning, escaped attack, although remaining in the house for weeks at a time. It was for the same reason that Dr. Rush was accustomed to advise families in the summer-time, not being able to leave the city, to cause their younger children especially, to spend their time above stairs. We have spent a lifetime ourselves in the West and extreme South, and know in our own person, and as to those who had firmness to follow our recommendation, that whole families will escape all the forms of Fall fevers who will have bright fires kindled at sunrise and sunset in the family room. But it is too plain a prescription to secure observance in more than one family in ten thousand. After the third frost, and until the Fall of the next year, it is an important means of health for persons to sleep with an outer door or window partly open, having the bed in such a position, as to be protected from a draught of air. We advise that no person should go to work or take exercise in the morning on an empty stomach; but if it is stimulated to action by a cup of coffee, or a crust of bread, or apple, or orange, exercise can be taken, not only with impunity, but to high advantage in all chill and fever localities.

---

"*Woman's Thoughts About Woman*," published by Messrs. Rudd & Carlton, New York, we have noticed twice before, and now again; because it is one of the few of the multitudinous issues of the season which merits a frequent perusal: its pages abound in practical sentiments, which are as solid as they are just. It is a book which will instruct and elevate every woman who reads it, and every man, as well.

